# Supplementary material for: Zoonotic Giardia duodenalis Genotypes and Other Gastrointestinal Parasites in a Badger Population Living in an Anthropized Area of Central Italy
Source: Pathogens. 2022 Aug 11;11(8):906. doi: 10.3390/pathogens11080906 (PMC9416481; doi:10.3390/pathogens11080906)
Supplement: Supplementary file 1 [file pathogens-11-00906-s001.zip › pathogens-1850720-supplementary.pdf]

Supplementary File S1 : Raw FASTA sequences (n=11) obtained from 10 badger stool samples (ID=Mm.n) at the ß-giardin (bg; number of sequences=8) and the triose phosphate isomerase (tpi; number of sequences=3) loci

>Mm.3_bg (identified as Assemblage A, sub assemblage AII)

TCCATAACGACGCCATCGCGGCTCTCAGGAAGGAGGCCCTCAAGAGCyTGAACGAyCTCGAGACGGGCATTGCCACG
GAGAACGCAGAAAGGAAGAAGATGTACGACCAGCTCAACGAGAAGGTCGCAGAGGGCTTCGCCCGCATCTCCGCCG
CGATCGAGAAGGAGACGATCGCCCGCGAGAGGGCCGTTAGTGCTGCCACGACAGAAGCGCTCACAAACACGAAGCT
CGTCGAGAAGTGCGTCAACGAGCAGCTCGAGAACGTCGCCTCGGAGATCCGCGCTATCCAGGAGGAGATCGACCGC
GAGAAGCCCGAGCGCAAGGA

>Mm.5_bg (identified as mixed Assemblage A and B infection)

CATAACGACGCCATCGCGGCTCTCAGGAAGGAGGCCCTCAAGAGCyTGAACGACCTCGAGACRGGCATyGCCACGGA
GAACGCMGARAGGAAGAAGATGTAYGACCAGCTCAACGAGAARGTCGCAGAGGGCTTCGCCCGCATCTCCGCCGCS
ATCGAGAAGGAGACGATCGCCCGCGAGAGGGCCGTYAGYGCTGCCACGACAGARGCSCTCACAAACACGAAGCTCGT
CGAGAAGTGCGTCAACGAGCAGCTCGAGAACGTCGCCTCGGAGATCCGCGCYATCCAGGAGGAGATCGACCGCGAG
AAGGCCGAGCGCAAGGA

>Mm.11_bg (identified as Assemblage A, sub assemblage AII)

TccATAACGACGCCATCGCGGCTCTCAGGAAGGAGGCCCTCAAGAGCYTGAACGAYCTCGAGACGGGCATTGCCACG
GAGAACGCAGAAAGGAAGAAGATGTACGACCAGCTCAACGAGAAGGTCGCAGAGGGCTTCGCCCGCATCTCCGCCG
CGATCGAGAAGGAGACGATCGCCCGCGAGAGGGCCGTTAGTGCTGCCACGACAGAAGCGCTCACAAACACGAAGCT
CGTCGAGAAGTGCGTCAACGAGCAGCTCGAGAACGTCGCCTCGGAGATCCGCGCTATCCAGGAGGAGATCGACCGC
GAGAAGGCCGAGCGCAaGGA

>Mm.14_bg (identified as Assemblage A, sub assemblage AII)

tcCATAACGACGCCATCGCGGCTCTCAGGAAGGAGGCCCTCAAGAGCyTGAACGAyCTCGAGACGGGCATTGCCACG
GAGAACGCAGAAAGGAAGAAGATGTACGACCAGCTCAACGAGAAGGTCGCAGAGGGCTTCGCCCGCATCTCCGCCG
CGATCGAGAAGGAGACGATCGCCCGCGAGAGGGCCGTTAGTGCTGCCACGACAGAAGCGCTCACAAACACGAAGCT
CGTCGAGAAGTGCGTCAACGAGCAGCTCGAGAACGTCGCCTCGGAGATCCGCGCTATCCAGGAGGAGATCGACCGC
GAGAAGGCCGAGCGCAAGGA

>Mm.16_bg (identified as Assemblage A, sub assemblage AII)

TCCATAACGACGCCATCGCGGCTCTCAGGAAGGAGGCCCTCAAGAGCCTGAACGAyCTCGAGACGGGCATTGCCACG
GAGAACGCAGAAAGGAAGAAGATGTACGACCAGCTCAACGAGAAGGTCGCAGAGGGCTTCGCCCGCATCTCCGCCG
CGATCGAGAAGGAGACGATCGCCCGCGAGAGGGCCGTTAGTGCTGCCACGACAGAAGCGCTCACAAACACGAAGCT
CGTCGAGAAGTGCGTCAACGAGCAGCTCGAGAACGTCGCCTCGGAGATCCGCGCTATCCAGGAGGAGATCGACCGC
GAGAAGGCCGAGCGCAaGGA

>Mm.22_bg (identified as Assemblage A, sub assemblage AII)

TcCATAACGACGCCATCGCGGCTCTCAGGAAGGAGGCCCTCAAGAGCCTGAACGAyCTCGAGACGGGCATTGCCACG
GAGAACGCAGAAAGGAAGAAGATGTACGACCAGCTCAACGAGAAGGTCGCAGAGGGCTTCGCCCGCATCTCCGCCG
CGATCGAGAAGGAGACGATCGCCCGCGAGAGGGCCGTTAGTGCTGCCACGACAGAAGCGCTCACAAACACGAAGCT
CGTCGAGAAGTGCGTCAACGAGCAGCTCGAGAACGTCGCCTCGGAGATCCGCGCTATCCAGGAGGAGATCGACCGC
GAGAAGGCCGAGCGCAAGGACGCAGACG

>Mm.23_bg (identified as Assemblage A, sub assemblage AII)

TcCATAACGACGCCATCGCGGCTCTCAGGAAGGAGGCCCTCAAGAGCCTGAACGAyCTCGAGACGGGCATTGCCACG
GAGAACGCAGAAAGGAAGAAGATGTACGACCAGCTCAACGAGAAGGTCGCAGAGGGCTTCGCCCGCATCTCCGCCG
CGATCGAGAAGGAGACGATCGCCCGCGAGAGGGCCGTTAGTGCTGCCACGACAGAAGCGCTCACAAACACGAAGCT
CGTCGAGAAGTGCGTCAACGAGCAGCTCGAGAACGTCGCCTCGGAGATCCGCGCTATCCAGGAGGAGATCGACCGC
GAGAAGGCCGAGCGCAAGGAC

>Mm.25_bg (identified as Assemblage A, sub assemblage AII)

CATAACGACGCCATCGCGGCTCTCAGGAAGGAGGCCCTCAAGAGCyTGAACGAyCTCGAGACGGGCATTGCCACGGA
GAACGCAGAAAGGAAGAAGATGTACGACCAGCTCAACGAGAAGGTCGCAGAGGGCTTCGCCCGCATCTCCGCCGCG
ATCGAGAAGGAGACGATCGCCCGCGAGAGGGCCGTTAGTGCTGCCACGACAGAAGCGCTCACAAACACGAAGCTCG
TCGAGAAGTGCGTCAACGAGCAGCTCGAGAACGTCGCCTCGGAGATCCGCGCTATCCAGGAGGAGATCGACCGCGA
GAAGGCCGAGCGCAAGGA

>Mm.20_tpi (identified as Assemblage A, sub assemblage AII)

GGCAAACACGTCAAAACAGTTGAGGATAGCAGCGCAGAATGTGTACCTAGAGGGGAACGGGGCGTGGACTGGCGA
GACAAGTGTTGAGATGCTTCAGGACATGGGTTTGAAGCATGTGATAGTAGGGCACTCTGAAAGACGCAGAATCATGG
GGGAGACCGACGAGCAAAGCGCCAAGAAGGCTAAGCGTGCCCTGGAAAAGGGGATGACGGTCATCTTCTGCGTCGG
AGAGACCTTGGATGAGCGCAAGGCCAACCGCACCATGGAGGTGAACATCGCCCAGCTTGAGGCGCTTGGCAAGGAG
CTCGGAGAGTCCAAGATGCTCTGGAAGGAGGTTGTCATTG

>Mm.15a_tpi (identified as Assemblage A, sub assemblage AII)

GtCCCTTCATCGGGGGTAACTtCAAGTGTAACGGCTCTCTTGACTTTATCAAGAGCCACGTGGCGGCAATTGCTGCCCA
TAAGATCCCTGATTCCGTGGACGTCGCCATTGCCCCCTCCGCCGTACACCTGTCAACAGCCACTGCGGCAAACACGTCA
AAACAGTTGAGGATAGCAGCGCAGAATGTGTACCTAGAGGGGAACGGGGCGTGGACTGGCGAGACAAGTGTTGAG
ATGCTTCAGGACATGGGTTTGAAGCATGTGATAGTAGGGCACTCTGAAAGACGCAGAATCATGGGGGAGACCGACG
AGCAAAGCGCCAAGAAGGCTAAGCGTGCCCTGGAAAAGGGGATGACGGTCATCTTCTGCGTCGGAGAGACCTTGGA
TGAGCGCAAGGCCAACCGCACCATGGAGGTGAACATCGCCCAGCTTGAGGCGCTTGGCAAGGAGCTCGGAGAGTCC
AAGATGCTCTGGAAGGAGGTTGTCATT

>Mm.16_tpi (identified as mixed Assemblage A and B infection)

tccgtggacgtTGTyGTTGCTCCCTCCGTTGTACACCTTTCTACAGCYATTGCGGCRAACAcTTCGAARyGTcTGArrAtaGCA
GCRCAGAAyGTGTAyCtGGAGggGAACGGkGCATGgaCyGGCGAGACAAgCGTyGAGAtGCTkCtGGACATGGGkyTGA
rsCATGTAATArTAGGaCACTcTGAAAGACGyAGAATCATGGGsGAGACCAAyGAGCArAgTGCyAaGAAGGCkAAGCGt
GCyCTGGAmAArGgkAtGACkGTyaTCTTyTGCrycGGAGAGACCCTGgATgaACGCAagGCCCAyaACaCTAtGgAGgTG
AAmATtgCTCAGCTTGAGgCTcTTAaCAAGGaGaTTGGAGAAtCmAAGAAgCTmtgGAAGAAGGTTgTAATTg
